# Dynamic logistic regression model and population attributable fraction to investigate the association between adherence, missed visits and mortality: a study of HIV-infected adults surviving the first year of ART

**DOI:** 10.1186/1471-2334-13-395

**Published:** 2013-08-27

**Authors:** Sylvia Kiwuwa-Muyingo, Hannu Oja, Ann Sarah Walker, Pauliina Ilmonen, Jonathan Levin, Mambule, Andrew Reid, Peter Mugyenyi, Jim Todd

**Affiliations:** 1 School of Health Sciences, University of Tampere, Tampere, Finland; 2 MRC Uganda Virus Research Institute, Entebbe, Uganda; 3 Department of Mathematics and Statistics, University of Turku, Turku, Finland; 4 Medical Research Council Clinical Trials Unit, London, UK; 5 ECARES, Université libre de Bruxelles, Brussels, Belgium; 6 Makerere University, Infectious Diseases Institute, Kampala, Uganda; 7 University of Harare, College of Health Sciences, Harare, Zimbabwe; 8 Joint Clinical Research Centre, Kampala, Uganda; 9 London School of Hygiene and Tropical Medicine, London, UK

**Keywords:** Dynamic logistic regression model, Population attributable fraction, ART, Adherence, Mortality, HIV

## Abstract

**Background:**

Adherence is one of the most important determinants of viral suppression and drug resistance in HIV-infected people receiving antiretroviral therapy (ART).

**Methods:**

We examined the association between long-term mortality and poor adherence to ART in DART trial participants in Uganda and Zimbabwe randomly assigned to receive laboratory and clinical monitoring (LCM), or clinically driven monitoring (CDM). Since over 50% of all deaths in the DART trial occurred during the first year on ART, we focussed on participants continuing ART for 12 months to investigate the implications of longer-term adherence to treatment on mortality. Participants’ ART adherence was assessed by pill counts and structured questionnaires at 4-weekly clinic visits. We studied the effect of recent adherence history on the risk of death at the individual level (odds ratios from dynamic logistic regression model), and on mortality at the population level (population attributable fraction based on this model). Analyses were conducted separately for both randomization groups, adjusted for relevant confounding factors. Adherence behaviour was also confounded by a partial factorial randomization comparing structured treatment interruptions (STI) with continuous ART (CT).

**Results:**

In the CDM arm a significant association was found between poor adherence to ART in the previous 3-9 months with increased mortality risk. In the LCM arm the association was not significant. The odds ratios for mortality in participants with poor adherence against those with optimal adherence was 1.30 (95% CI 0.78,2.10) in the LCM arm and 2.18 (1.47,3.22) in the CDM arm. The estimated proportions of deaths that could have been avoided with optimal adherence (population attributable fraction) in the LCM and CDM groups during the 5 years follow-up period were 16.0% (95% CI 0.7%,31.6%) and 33.1% (20.5%,44.8%), correspondingly.

**Conclusions:**

Recurrent poor adherence determined even through simple measures is associated with high mortality both at individual level as well as at the ART programme level. The number of lives saved through effective interventions to improve adherence could be considerable particularly for individuals monitored without using CD4 cell counts. The findings have important implications for clinical practice and for developing interventions to enhance adherence.

## Background

Adherence is one of the most important determinants of viral suppression among people receiving antiretroviral therapy (ART) for HIV-infection; conversely poor adherence is associated with acquired antiretroviral drug resistance [1-4]. Adherence measurements and methods for summarizing adherence vary depending on resources, with some researchers showing that adherence may be seen as a stochastic process [5-7]. Adherence has been measured using different indices such as drug possession ratio [8], or simple proportions or counts of missed pills/appointments [8-11], or measured using electronic medication monitors [12]. Although good adherence is essential for the success of ART, there is no gold standard or benchmark measure of adherence. However, electronic monitoring of adherence was more strongly associated with viraemia compared to self-reported adherence monitoring [13,14]. Other studies did not show any difference between adherence measures and viral load suppression [15].

For HIV-infected adults, poor adherence leads to low levels of drug, leading to viral replication, drug resistance, and viral rebound. That in turn causes CD4 decline which leads to morbidity/mortality. Several studies have demonstrated the association between adherence and mortality [16,17]. However, factors including drug toxicity/side effects, resistance, disease stage and sociodemographic factors may be associated with both adherence and mortality either being on the causal pathway between adherence and mortality (as intermediate factors), or as a proxy for some unmeasured confounding depending on the time order of the events.

Many studies [18] have identified prognostic factors for mortality following ART initiation; the most recent values of immunological and virological factors are typically the most predictive. In contrast a single report of poor adherence may not be associated with immediate mortality, but rather recurrent poor adherence, i.e. adherence history, may be the main driver of longer-term mortality. Dynamic logistic regression can be used to examine the delayed effect of predictors on death, which may be seen several months after the reported poor adherence, in a similar way to a time-dependent Cox model. However, the advantage of dynamic logistic regression is that it easily allows estimation of the achievable effects of risk factor manipulation at the population level, i.e. the proportion of deaths that could have been avoided with optimal adherence.

Here we examine the association between mortality and adherence to ART using data from the DART (Development of AntiRetroviral Therapy) trial in Uganda/Zimbabwe [19], in which participants were randomly assigned to laboratory and clinical monitoring (LCM) or clinically driven monitoring (CDM) of ART. Our main aim was to investigate the effect of adherence history on the risk of death in the second year on ART onwards at the individual level (odds ratios, OR) and at the population level (population attributable fraction, PAF). We also assess the time delay in the effect of adherence on the risk of mortality. Over 50% of all deaths in the DART trial occurred during the first year on ART, when the risk of death is higher, and mortality may be less influenced by adherence [19,20]. In this paper we focussed on participants surviving the first year of ART, in order to investigate the impact of longer-term adherence to treatment on mortality.

## Methods

### Data and problem

#### Study population

3316 ART-naive adults with symptomatic HIV disease and CD4 <200 cells/ *μ*L were enrolled in the DART trial January 2003-October 2004 at three centers, 2 in Uganda (and 1 satellite) and 1 in Zimbabwe, and initiated triple drug ART [19]. 2469(74%) received combivir (coformulated zidovudine-lamivudine) and tenofovir disoproxil fumarate, 300 received combivir and abacavir, and 547 received combivir and nevirapine as their first-line ART. Participants were randomly assigned to laboratory and clinical monitoring (LCM) or clinically driven monitoring (CDM); both groups saw a physician and had a routine full blood count with lymphocyte subset CD4 every 12 weeks, but total lymphocyte or CD4-cell counts were not returned for the latter group. All participants were reviewed by a nurse every 4 weeks (using a standard symptom checklist) and had 4-weekly ART refills.

**Figure 1:**
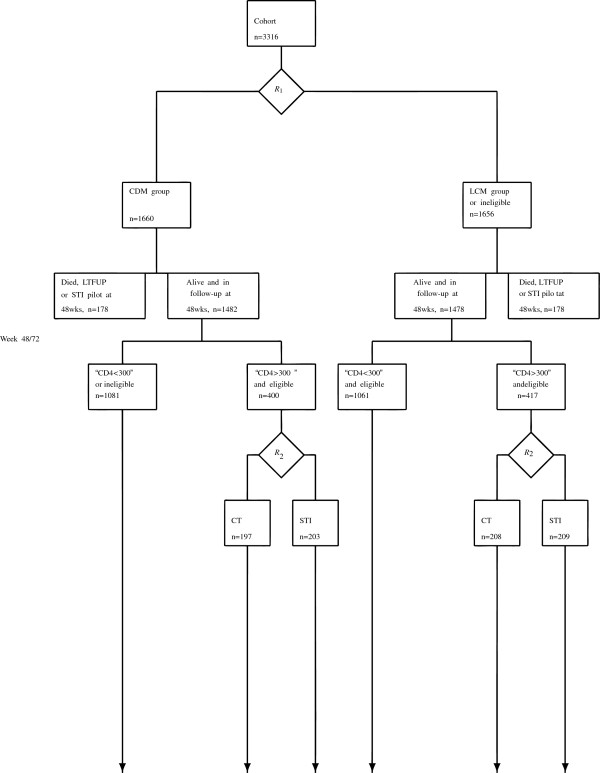
**Illustration of the first and second randomization of the participants of the DART trial (356 died/lost to follow-up or excluded in first year).** The latter confounds adherence measurements: 813 good early response participants were randomized between continuous therapy (CT) and cycles of 12 weeks on/off ART structured treatment interruptions (STI). We analysed all participants alive and in follow-up at 48 weeks.

Adherence behaviour was potentially confounded by a further partial factorial randomization comparing structured treatment interruptions (STIs, cycles of 12 weeks on/off ART) with continuous ART (CT) in participants with good early response (CD4 >300 cells/ *μ*L) at the week 48 or 72 CD4 count [21] (This followed a small pilot study in 137 patients; these pilot patients were excluded from all analyses). The CT/STI randomizations were stopped early, in March 2006, due to inferiority of STIs, and all patients randomized to STIs returned to continuous treatment. (See Figure 1.) The potential confounding effect of STI is addressed in the model in the following way: In our dynamic modeling, the effect of adherence on mortality is obviously not confounded by the STI group before the randomization. After the randomization the individuals randomized to the STI group are parameterized as a separate group from those remaining on continuous therapy with known adherence behaviour, and therefore they do not confound the main effect of adherence on mortality after the randomization either. We do not wish to censor these individuals at the time of STI randomization as they may still carry information about the connection between other explanatory variables and mortality.

Participants were followed under their randomized strategies until December 31, 2008. More than 50% of the total deaths over a median 5 years follow-up in DART occurred during the first year on ART, >25% in the first 3 months [20]. These deaths in the first year on ART are plausibly due to carryover effects from the advanced immunosuppression prior to at ART initiation (ART cannot work fast enough) and therefore are less likely to be related to ART adherence. Including these deaths in analysis may therefore dilute the impact of adherence over the longer-term. As our focus was on the impact of adherence on longer-term mortality on ART, we therefore excluded the 171 participants that died and the 48 lost to follow-up during the first year on ART, leaving 2960 participants alive and in follow-up at one year for analysis, the same dataset as previous analysis [7,22]. This allowed us to investigate adherence history measures based on up to 12 previous visits.

#### Informed consent

Individual informed consent was obtained from each participant at screening and, if eligible, at enrolment for randomisation to (i) the two monitoring strategies (LCM, CDM)(all participants) and (ii) STI or CT if eligible.

#### Ethics statement and approval

DART received ethics committee approval in Uganda from the Science and Ethics Committee (SEC), in Zimbabwe from Medical Research Council of Zimbabwe (MRCZ) which is the National Ethics Committee (NEC) and the Imperial College Research Ethics committee in the UK. The trial was registered (ISCRTN13968779).

#### Adherence measurements

Participants’ ART adherence was assessed by pill counts and a structured questionnaire administered at each scheduled 4-weekly clinic visit [23]. The questionnaire asked whether they had missed any doses in the last month, were late for the visit, had forgotten to take any doses at the weekend, or missed any ART on the previous 4 days. Adherence data is missing if (i) the participant missed his/her visit (“missing”), or if (ii) the participant attended the visit but did not, for some unknown reason, respond to these questions (“non-response”). We consider patients who had been randomized to STI as an additional class of missing (ie as a separate group), since their adherence measurements and outcomes may no longer be directly comparable with those taking ART continuously.

#### Outcome and problem

Our primary outcome is mortality in those surviving the first year on ART, censored at the minimum of their last follow-up visit or 31 December 2008. We model the effect of adherence history on the risk of death at the individual level (odds ratios from a fitted dynamic logistic regression model) and at the population level (population attributable fraction, PAF) using the estimated model parameters. We also assess the time delay in the effect of adherence on the risk of mortality. As the main trial results demonstrated a small but statistically significant difference in mortality between the LCM and CDM groups [19], analyses were done separately for both groups, and estimates adjusted for relevant pre-ART confounding factors.

### Statistical methods

#### Dynamic logistic regression model

The data are analyzed using a dynamic logistic regression model. The follow-up period of each individual is divided into successive 4-weekly periods between the clinic visits. Then the probability of dying in a certain period is the probability of surviving previous periods multiplied by the conditional probability of dying in that period conditional on previous survival. The model is then the regular logistic model for these conditional probabilities and the explanatory variables for death in a period may include any variables describing the individual’s history up to the beginning of the period. The odds ratios are then for the instantaneous risk of dying in a period. The model may be seen as a discrete time version of the Cox’s proportional hazard model (details given in Additional file 1: appendix A).

#### Population attributable fraction (PAF)

The estimated model is used to find an estimate for population attributable fraction as follows. For each individual with known covariate history, one can, with an estimated model, estimate the probabilities of dying on each 4-weekly interval in an imaginary case of his or her optimal adherence. If this is done separately for each individual in a population, the results can be summarised at the population level as a survival curve for an imaginary population of patients with optimal adherence. If this curve is compared to the observed overall survival curve (or the curve without the manipulation), one can calculate an estimate for the proportion of deaths that could have been avoided by manipulation, that is, the population attributable fraction of deaths due to non-optimal adherence. (See Additional file 2: appendix B for more details).

#### Summarising adherence history (***x***_***1***_)

Here we use the 4-weekly question “missed a dose in the last month”, because it was most strongly associated with viral load [23]. At each visit *t*=1,2,... the adherence variable can take the following four values 

“poor”, “good”, “non-response”, and “missing”,

where “poor” and “good” mean “missed a dose in the last month” and “did not miss a dose in the last month”, respectively.

The history of adherence behaviour up to the visit *t* is then summarized as follows. First, we consider the visits *t*−8,*t*−7,...,*t*−3 (6 visits, that is, 6 months) to calculate the following three independent indicator variables 

“poor at least once”, “non-response at least once”, “missing at least once”,

for visits *t*. These three adherence variables are not mutually exclusive, that is, any combination of zeros and ones is possible. Whilst this is only one way to combine 4-weekly adherence measurements, it is a simple summary which retains much of the historical information. As a delay in the effect of adherence on mortality is likely given the causal pathway (see above), we used adherence data from visits *t*−8,*t*−7,...,*t*−3 only to predict the mortality between visits *t*−1 and *t*. The adherence measurements at visits *t*−2 and *t*−1 may then be seen rather as intermediate factors between adherence history and death. In fact, preliminary analysis showed that patients dying between visits *t*−1 and *t* often missed visits *t*−2 and *t*−1 for reasons that were clearly more related to their mortality risk than their adherence behaviour. Whilst several other time intervals *t*−*u*,...,*t*−*v* were considered, we presented results only for *t*−8,*t*−7,...,*t*−3 as the associations with mortality were generally very similar. One can naturally also try other time intervals *t*−*u*,...,*t*−*v* with different choices of *u* and *v* but then the incidence and interpretation of “poor at least once”, for example depends strongly on the length *u*−*v*+1. The comparison of different fitted models is therefore difficult, and we tried to keep the model as simple as possible.

The adherence measurement are, however, confounded by the CT/STI randomization as explained above. We therefore need a fourth indicator 

“randomized to STI”

which is 1 at visit *t* if the patient has been randomized to STI before visit *t*. If 1, then the three adherence indicators above lose their interpretation and are all set to be 0. Essentially therefore after having been randomised to STI, patients are modelled separately both in terms of their subsequent on-ART adherence and its relationship to mortality. If all four indicators get the value zero, then the patient is not randomized to STI and adherence behaviour is “optimal”; this is thus the reference class in the modeling.

#### Confounding variables (***x***_***2***_)

We fitted an adjusted model including the following fixed pre-ART characteristics: age (18-35, 35-50, 50+), sex (male, female), WHO disease stage (2, 3, 4), body mass index (BMI) (-20, 20-27, 27+), and CD4 cell count (0-49, 50-99, 100-149, 150-199), categorizing continuous variables to allow for non-linearity.

We did not treat, for example, time-dependent CD4 cell count as a confounding variable. We rather considered it as an intermediate variable, meaning that CD4 cell count at time *t* is likely affected by previous adherence history, and is simultaneously a subsequent predictor of mortality. For this reason, only pre-ART CD4 count was included as a baseline factor (similarly for WHO disease stage and BMI). Although follow-up and adherence history in this study starts at 12 months, we did not adjust for CD4 count 12 months after starting ART for the same reason, namely adherence behaviour during the first 12 months could have already had an impact on CD4, which in turn would have an effect on mortality. The main exposure of interest is adherence history and adherence measurements were taken on the preceding nine months in our analysis (ie adherence history from 3-9 months on ART for the first interval). To adjust for the effect of time on ART, we included a variable follow-up time, categorized as (1,2], (2,3], (3,4], (4,5], and (5,6] years on ART.

## Results

### Mortality risk at individual level

**Figure 2:**
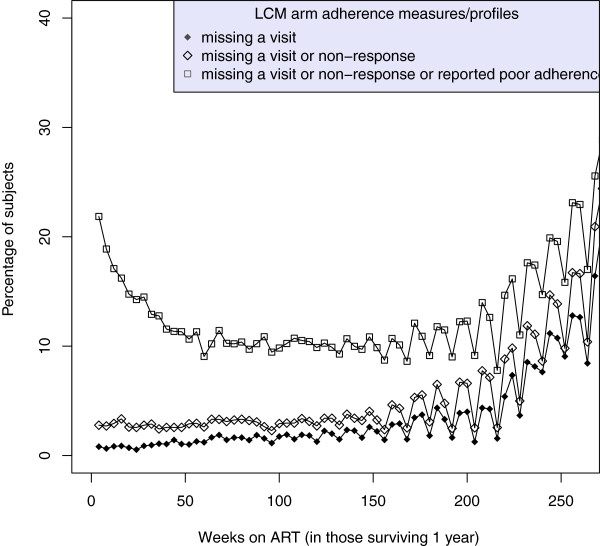
**Proportion of participants at weeks *t=52,56,60,… *on ART in the LCM group who (i) miss a visit (solid circle curve) (ii) miss a visit or do not respond to the adherence question (diamond symbol curve), (iii) miss a visit, do not respond, or report poor adherence (square symbol curve).** The periodicity in the curves after 3 years is partly due to a small group of patients who moved to 12-weekly ART refills and therefore missed intervening 4-weekly visits.

**Figure 3:**
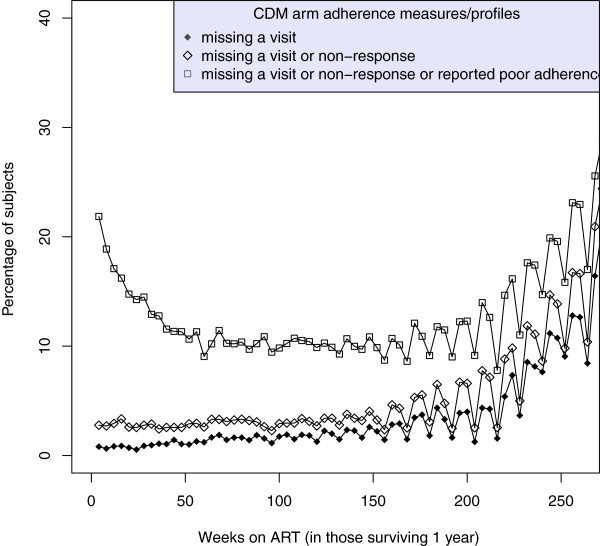
**Proportion of participants at weeks *t=52,56,60,… *on ART in the CDM group who (i) miss a visit (solid circle curve) (ii) miss a visit or do not respond to the adherence question (diamond symbol curve), (iii) miss a visit, do not respond, or report poor adherence (square symbol curve).** The periodicity in the curves after 3 years is partly due to a small group of patients who moved to 12-weekly ART refills and therefore missed intervening 4-weekly visits.

Median (IQR) follow-up after 1 year on ART in the 2960 patients surviving 12 months was a further 3.9 (3.5-4.3) years on ART, see Table 1. The proportion of individuals reporting good adherence at each visit remained very high and stable over most of the five year period (Figure 2 and Figure 3), with little difference in the adherence profiles between the LCM and CDM groups. However, the number of missing visits did increase with time, at least in part because, after approximately 3 years on ART, a small number of participants moved to 12-weekly visits, with telephone nurse visits in-between (without adherence data). This may result in a small bias in the estimates of the effect of missing visits on mortality.

**Table 1:** Characteristics of ART naive adults initiating ART in Uganda and Zimbabwe and surviving the first year of Therapy by main randomisation arm

	**Baseline characteristics at**	
	** randomisation and week 48**	
**Randomisation in main DART trial**	**LCM**	**CDM**
**Total participants**	**1478**	**1482**
Sex		
Female	970(66%)	952(64%)
WHO stage		
2	331(22%)	291(20%)
3	827(56%)	844(57%)
4	320(22%)	345(23%)
BMI		
<20	489(33%)	515(35%)
20-27	846(58%)	831(56%)
>27	130(9%)	127(9%)
CD4 cells/ *μ*L		
Median (IQR)	84(33-138)	86(31-140)
Age(years) at ART initiation		
Median (IQR)	37(32-42)	36(32-42)
18-35	589(40%)	593(40%)
35-50	792(54%)	796(54%)
>50	97(6%)	93(6%)
Initial ART regimen		
Tenofovir (TDF)	1075(73%)	1087(73%)
Nevirapine (NVP)	260(18%)	251(17%)
Abacavir (ABC)	143(10%)	142(10%)
Follow up at 48 weeks		
STI/CT randomisation		
Not randomised	1061(72%)	1082(73%)
Randomised to STI	209(14%)	203(14%)
Randomised to CT	208(14%)	197(13%)
CD4 cells/ *μ*L at week 48		
Median (IQR)	201(139-283)	200(141-280)
Weight at week 48		
Median(IQR)	63(57-71)	63(56-71)

As previously reported [19], patients randomized to CDM had a higher mortality risk than LCM patients with an unadjusted odds ratio 1.56 (95% CI 1.17,2.08) after the first year on ART. We therefore did the analyses separately for both groups.

The estimated odds ratios (OR) with 95% CI for different risk factors for mortality from the dynamic logistic model in LCM and CDM groups are given in Tables 2 and 3, respectively, and demonstrate a clear association between poorer adherence and 4-weekly probability of dying in the CDM group. In the LCM group the difference in mortality risk between poor and optimal adherers was in the same direction but was non-significant. The adjusted odds ratios (with p values) for “poor at least once vs. optimal”, “non-response at least once vs. optimal” and “missing at least once vs. optimal” were 1.30 (0.3), 1.98 (0.03) and 3.60 (<0.001) in the LCM group and 2.18 (<0.001), 2.09 (<0.001) and 3.65 (0.005) in the CDM group. The largest differences between LCM and CDM groups can be seen in the odds ratios for poor vs. optimal (1.30 and 2.18 for LCM and CDM groups, correspondingly). However, 95% CI were relatively wide, and fitting both groups together in a single model, a heterogeneity test did not provide evidence of difference (p >0.1). There was strong evidence of heterogeneity in the 3 adherence estimates within each model ie the impact of poor adherence on mortality risk differs from impact of non-response differs from the impact of missing (p <0.001). The adjusted odds ratios for “having previously been randomized to STI vs. optimal” (technical indicator that at least partially addresses confounding by the CT/STI randomization) in the LCM and CDM groups were 0.86 (0.29, 2.05) and 2.07 (1.03, 3.85). This indicates that over the longer-term those previously undergoing STIs in the CDM group still had poorer outcomes, likely due to delays in identifying first-line drug failure in the CDM group [19].

**Table 2:** Estimated unadjusted and adjusted odds ratios (OR) with 95 percent confident intervals for the risk of death obtained from the dynamic logistic regression model and based on 1478 individuals in the LCM group alive after the first year of follow-up

	**Unadjusted OR**	**95% CI**	**Adjusted OR**	**95% CI**	**P-value**
Adherence history:					
poor at least once vs optimal	1.34	(0.81, 2.17)	1.30	(0.78, 2.10)	0.30
non-response at least once vs optimal	1.99	(1.01, 3.63)	1.98	(1.00, 3.62)	0.03
missing at least once vs optimal	3.26	(1.65, 5.96)	3.60	(1.80, 6.65)	<0.001
Pre-ART WHO disease stage					
stage 2	1(ref)		1(ref)		0.8
stage 3	1.08	(0.61, 2.02)	1.02	(0.57, 1.91)	
stage 4	1.48	(0.76, 2.92)	1.19	(0.60, 2.41)	
Pre-ART CD4 cell count					
0-49	1.69	(0.88, 3.51)	1.43	(0.72, 3.07)	
50-99	1.27	(0.61, 2.77)	1.18	(0.55, 2.61)	
100-149	1.07	(0.49, 2.41)	1.01	(0.45, 2.29)	
150-199	1(ref)		1(ref)		0.6
Pre-ART Body mass index					
<20	1.35	(0.82, 2.18)	1.31	(0.79, 2.14)	
20-27	1(ref)		1(ref)		0.5
>27	1.37	(0.59, 2.79)	1.33	(0.57,2.77)	
Age at ART initiation groups					
18-35	1(ref)		1(ref)		0.9
35-50	0.84	(0.53, 1.35)	0.92	(0.57, 1.50)	
50+	0.89	(0.31, 2.09)	1.03	(0.35, 2.48)	
Sex					
Female vs Male	1.23	(0.76, 2.04)	1.31	(0.79, 2.22)	0.3
Time since ART initiation in years					
≤2	1(ref)		1(ref)		0.06
2-3	0.37	(0.17, 0.73)	0.40	(0.18, 0.79)	
3-4	0.57	(0.30, 1.04)	0.60	(0.31, 1.10)	
4-5	0.49	(0.24, 0.93)	0.47	(0.23, 0.91)	
>5	0.76	(0.34, 1.54)	0.65	(0.29, 1.34)	

Adherence history is given by 4 time-dependent indicators with “optimal” adherence as a reference class.

**Table 3:** Estimated unadjusted and adjusted odds ratios (OR) with 95 percent confident intervals for the risk of death obtained from the dynamic logistic regression model and based on 1482 individuals in the CDM group alive after the first year of the follow-up

	**Unadjusted OR**	**95% CI**	**Adjusted OR**	**95% CI**	**P-value**
Adherence history:					
poor at least once vs optimal	2.14	(1.45, 3.14)	2.18	(1.47, 3.22)	<0.001
non-response at least once vs optimal	2.16	(1.29, 3.52)	2.09	(1.22, 3.40)	<0.001
missing at least once vs optimal	3.46	(2.07, 5.54)	3.65	(2.15, 5.92)	0.005
Pre-ART WHO disease stage					
stage 2	1(ref)		1(ref)		0.51
stage 3	1.67	(0.99, 3.03)	1.39	(0.82, 2.55)	
stage 4	1.61	(0.88, 3.08)	1.28	(0.68, 2.48)	
Pre ART CD4 cell count					
0-49	3.61	(1.88, 7.85)	3.43	(1.74, 7.62)	
50-99	2.75	(1.36, 6.14)	2.62	(1.28, 5.90)	
100-149	2.49	(1.20, 5.63)	2.45	(1.18, 5.57)	
150-199	1(ref)		1(ref)		0.01
Pre-ART Body mass index					
<20	1.63	(1.12, 2.38)	1.33	(0.90, 1.96)	
20-27	1(ref)		1(ref)		0.35
>27	0.98	(0.43, 1.93)	1.07	(0.46,2.15)	
Age at ART initiation groups					
18-35	1(ref)		1(ref)		0.07
35-50	0.72	(0.49, 1.05)	0.83	(0.56, 1.24)	
50+	1.45	(0.74, 2.63)	1.74	(0.87, 3.21)	
Sex					
Female vs Male	0.62	(0.43, 0.90)	0.72	(0.49, 1.06)	0.10
Time since ART initiation in years					
≤2	1(ref)		1(ref)		0.13
2-3	1.65	(0.98, 2.84)	1.66	(0.98, 2.86)	
3-4	1.10	(0.61, 1.99)	1.10	(0.61, 1.98)	
4-5	1.64	(0.96, 2.86)	1.56	(0.90, 2.74)	
>5	0.91	(0.38, 1.96)	0.80	(0.33, 1.73)	

Adherence history is given by 4 time-dependent indicators with “optimal” adherence as a reference class.

For those monitored following LCM, mortality risks seemed somewhat lower at visits either 2-3 or 4-5 years on ART (p=0.06), none of the other pre-ART factors were significantly associated with mortality risk after 1 year on ART (p >0.3). In contrast, in the CDM group the post-1-year mortality risks were higher for patients with pre-ART <150 cells/ *μ*L (p=0.01 for categorical pre-ART CD4 variable), and for patients aged >50 years at ART initiation (p=0.07 for categorical variable age). There was no additional effect of regimen at ART initiation with the adjusted OR for Nevirapine, Abacavir versus Tenofovir 1.18 (0.61, 2.12), 1.21(0.52, 2.45), p=0.8 in LCM and 0.52 (0.25, 0.96), 0.64 (0.28, 1.24), p=0.1 in CDM. All these results were consistent with the main trial results of DART [19], which showed that differences in mortality between LCM and CDM were most likely due to delayed switching (for failure of first-line to second-line treatment) in the latter group without CD4 count monitoring. Low pre-ART CD4 and higher age have been previously identified as factors which increase the risk of ART failure. We found no evidence that the impact of current adherence varied according to pre-ART CD4 (p=0.4).

To consider the bias caused by the participants who moved to 12-weekly visits (with telephone nurse visits in-between), we also fitted interactions between time indicator (4,6] and adherence variables. There was no statistically significant interactions in the LCM group. The only statistically significant interaction term (p=0.004) in the CDM group was for the interaction between non-response and time. The adjusted odds ratio for non-response vs. optimal then changed from 3.72(95% CI 2.07,6.35) to 0.10(95% CI 0.01,0.38). This change may be due to the fact that the subjects with 12-weekly visits are classified as non-responders but in fact they are good adherers and therefore eligible for 12-weekly refills. In further analyses, we also considered censoring individuals with 12 weekly telephone visits from the 4th year. The estimates for the main exposure adherence variables and PAF censoring the 12-weekly telephone visits were very similar to the analysis including these participants in both LCM and CDM groups.

### Population attributable fraction

**Figure 4:**
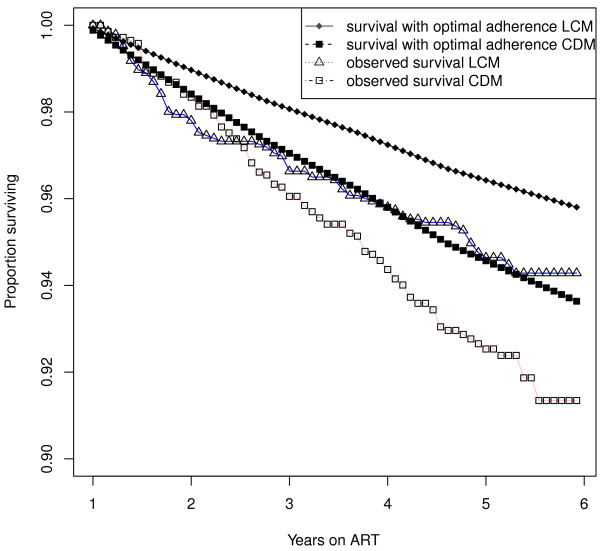
Estimated (weighted Kaplan-Maier) survival curves and estimated survival curves under optimal adherence in the LCM and CDM groups.

In Figure 4 estimated survival curves are shown in the LCM and CDM groups. In both groups, we calculated a weighted Kaplan-Maier survival curve estimate for the original population assuming all patients had been intended to take ART continuously (ie if the CT/STI randomization had not occurred), by using weights 2 and 0 for those randomized to CT and STI respectively (after randomization), and 1 for non-randomized patients (and before STI/CT randomisation). Then we used the model-based hypothetical estimated survival curves for the same population but with optimal adherence history (all four indicators constantly zero, confounded variables as in the population) were calculated. The PAF estimates are then based on the values of these curves at 5 years.

The estimated proportion of deaths that could have been delayed (by eliminating exposure to non-optimal adherence) 1-6 years after initiating ART was 16.0% among patients receiving LCM, but was higher at 33.1% in CDM. The corresponding 90% confidence intervals based on 200 bootstrap samples were (-0.7%,31.6%) and (20.5%,44.8%), respectively. These proportions correspond to absolute increases in 6-year survival (5 years after our 48 week baseline) of 1% in the LCM group (from 94% observed to 95% with optimal adherence) and 3% in the CDM group (from 91% observed to 94% with optimal adherence), with corresponding death rates from 1.2/100 to 1/100 person years in LCM and 1.8/100 to 1.2/100 person years in CDM.

## Discussion

The results of this study emphasize the importance of ART adherence for reducing long-term mortality risk in HIV-infected adults taking ART. Three measures of non-optimal adherence, missing a dose, not responding to the adherence questionnaire, and missing a visit at least once during a preceding 6 month period, independently predicted increased immediate mortality risk in patients only monitored using clinical symptoms in the DART trial in Uganda and Zimbabwe, with the latter two measures also predictive in patients being monitored using CD4 cell counts. There was strong evidence within each model that the impact of poor adherence on long-term mortality risk differs from non-response, and differs from those with missing response. These effects were independent of potential pre-ART confounders WHO disease stage, CD4 cell counts, body mass index, age, and sex, and time-dependent STI group and time on ART. Whilst there was a non-significant trend towards a larger effect of poor adherence in the group of participants monitored without CD4 cell counts, perhaps the most important finding was that optimal adherence with clinical monitoring seemed to provide as good overall survival outcomes as those actually observed in the group receiving CD4 monitoring in the trial (Figure 4).

Although the survival under optimal adherence is unknown in this context, the major advantage of the dynamic logistic regression model is that it can be estimated. Only modest absolute differences in mortality risk were observed (1% in LCM and 3% in CDM at 6 years from ART initiation), but the estimated proportions of deaths that could have been delayed (by eliminating non-optimal adherence) within 1-6 years after initiating ART were remarkably high in both groups - 16% in the LCM and 33% in CDM. This equates to absolute increases in 6-year survival from 94% observed to 95% with optimal adherence in LCM and from 91% observed to 94% in CDM. The differences in estimated mortality between LCM and CDM under optimal adherence were narrower than observed in the trial itself, suggesting that, as well as its role in detecting ART failure earlier, one major role of CD4 monitoring could be to to reinforce good adherence behaviour, or to identify those with adherence issues. This may be particularly valuable if laboratory monitoring can be integrated with adherence data to focus interventions on priority patients who need more support. However, too few deaths were observed to rule out the possibility that differences in associations between adherence and mortality in LCM and CDM were due to chance alone.

Our main study findings are based on the association between adherence in the 3-9 month preceding interval and immediate risk of mortality. Preliminary investigation suggested that nominal adherence behaviour in the immediately preceding intervals was strongly subject to reverse causality (ie sick patients do not attend visits), and hence we did not include adherence at the immediately preceding intervals in our adherence history variable. Whilst several other time intervals *t*−*u*,...,*t*−*v* were considered, we presented results only for the period 3-9 months before as the associations with mortality were generally very similar across that time period. Other adherence summaries could also have been considered eg ’poor at least once in 6 months’, for example, could have been replaced by ‘poor at least k times in *u*−*v*+1 months’, but having shown strong associations with the simplest model formulation, additional complexity is unlikely to have improved models substantially, and the population attributable fraction (PAF) is unlikely to change much.

The strongest adherence predictor of immediate mortality was missing one or more visits in the 3-9 month preceding interval, with a remarkably strong effect in both monitoring groups. DART participants were provided with ART until their next scheduled clinic visit, and so missing a visit typically represented running out of drugs (with the exception of those on 12-weekly ART re-fills in the latter part of the trial). Such interruptions may lead to development of drug resistance [24], which would promote virological failure and hence CD4 cell count failure, and hence increased risk of mortality. Alternatively, they could identify a group of patients with poorer health-seeking behaviour; delays in seeking healthcare could increase the risk of dying following any morbid events. Actively seeking high risk patients who have missed clinic appointments might have a significant impact on mortality. There is an urgent need for more studies on patients who missed clinic appointments, the causes of such missed appointments, and the outcomes experienced by these patients, especially in places where laboratory monitoring is not available. Although not statistically significant, there was also a suggestion that being randomised to STI may have increased the mortality risk of CDM participants more than LCM participants. Interestingly, no mortality difference was observed during the randomised follow-up on STI vs CT (5 vs 4 deaths respectively) and there was no evidence of interaction with monitoring strategy during this time [21]. One possible explanation is that STIs raised the risk of first-line ART failure. So the later detection of first-line failure in CDM could have led to STI being associated with increased mortality risk in CDM but not LCM subsequently during the trial. Similarly, other pre-ART factors, such as pre-ART CD4 and older age, were also associated with ART failure and with increased mortality risk in CDM, but not LCM.

Several studies have assessed the impact of adherence on mortality and report evidence of an association between adherence and long-term mortality risk among HIV infected individuals receiving ART. For example, Chi *et al* reported a 1.7 fold increased risk of post 12 month mortality in a large scale public sector HIV care programme in Zambia in those with <80% drug possession ratio (DPR) based on pharmacy refill [16]. Lima *et al*[17] demonstrated a 3 fold increased risk of mortality for a DPR adherence threshold of <95%; Nachega *et al*[25] reported a 3 fold increased risk of mortality in a South African private sector HIV care programme for pharmacy claims adherence <80%. Like our study, most studies used indirect methods of adherence assessment based on self-reports, rather than electronic medication monitoring which is expensive and intrusive, although provides qualitatively and quantitatively different information about adherence behaviours [10]. One challenge in assessing the association between adherence and mortality is that the impact of poor adherence may also be associated with the length of follow up. Another challenge is that adherence may be confounded with other lifestyle and health-seeking behaviours which might also impact outcomes. For example, a meta analysis of adherence to drug/placebo showed reduced mortality associated with good adherence to both active drug regimens and to placebo [26]. Thus participants with good adherence to study drugs may also have better behaviours (eg diet, exercise) and more regular follow-up which may affect their outcome. Another challenge is assessing the time between poor adherence and death, and that several other events may happen during that time period, such as increased viral load, lowered CD4 counts, changing drug regimes and opportunistic infections. This analysis using dynamic logistic regression model enabled us to assess the full effect of adherence on mortality without confounding from factors on the causal pathway. Nevertheless these studies, and others assessing adherence-mortality relationship, demonstrate that adherence remains important in reducing mortality risk among HIV infected individuals. Our finding that missed visits (also a proxy for the DPR since missing visits typically means running out of drugs) are closely associated with higher longer-term mortality risk, sets the stage for future studies to address causal relationships between adherence and mortality but more importantly, to quantify, for patients and policy makers, the impact of taking drugs and/or missed visits.

A potential limitation of our study was that many patients were on triple nucleoside reverse transcriptase inhibitor (3NRTI) regimens which are no longer recommended in WHO guidelines. However, previous analyses of adherence during the first-year on ART found similar associations between self-reported missing doses in the last month and VL suppression [23] as found in those receiving standard WHO-recommended regimens, suggesting that results may well be generalizable. Further, the typically lower level viral load suppression found with 3NRTI regimens might in fact lead to clearer associations between poor adherence and mortality over shorter timescales than might be observed with more robust regimens. Whilst our inclusion only of those surviving 48 weeks on ART might be considered a limitation, we would rather regard it as an advantage, since factors influencing early mortality on ART are much more likely driven by pre-ART experiences and analyses including both early and late deaths may therefore dilute the impact of differences associated with adherence over the longer-term. As with all observational analyses, we also make the assumption of no unmeasured confounders. Our model is also unable to address the role of time-dependent confounding, eg from current CD4 count, which would require the use of more sophisticated causal models. However, as adherence to ART precedes immunological recovery, our analysis adjusting only for baseline (pre-ART) factors is still able to estimate the overall impact of adherence. Another limitation was that we used self-reported measurements of adherence, which generally overestimate adherence [14] as they are subject to recall and/or social desirability bias [27]. Nevertheless, many studies have shown at least some association between self-reported and electronically monitored adherence, and have also shown associations between some self-reported measures and viral load suppression, suggesting self report has some clinical significance [4,15,24]. Self-report adherence measures are also preferred in many clinical settings for their simplicity and practical use. Whilst here we used such a self-report measure, it was also most strongly associated with viral load suppression in an earlier DART study [23]. “Optimal” adherence levels (proportion not missing a dose in the last month, ie >95%, see Figure 2 and Figure 3) are similar to, or even higher than, those reported by several other clinical trials (>80%) [10]. Even if this measure overestimates true adherence, it was nevertheless strongly associated with mortality, indicating that the effect of true adherence would likely be even greater if this could have been measured using other more accurate adherence measures, such as electronic monitoring devices such as MEMSCAPS. Other summary adherence measures could be considered rather than a dichotomous adherence measure; our study shares this limitation inherent in many studies trying to explore explanatory factors that might translate into clinical use within busy ART clinics. One strength of our study is that we explicitly considered missing visits and non-response as different types of behaviour to reporting non-adherence - this also avoided the need to make other assumptions about missing data.

## Conclusions

In summary, we show that poor adherence to ART is associated with increased mortality risk at the individual level, and that the estimated proportions of deaths on long-term ART that could be delayed at population level (by eliminating non-optimal adherence) are similar to benefits from CD4 cell count monitoring of ART and thus high enough to warrant considerable efforts on interventions to support and improve adherence at a programme level, particularly for patients not being monitored using CD4 counts or viral loads. One mechanism through which laboratory monitoring may improve outcomes appears to be to mitigate some of the negative consequences of poor adherence by identifying poor adherers earlier and enabling interventions. Several past and recent studies suggest that several effective interventions that improve ART adherence exist [28-30]. However the effects are small and also transient - there is no simple strategy for intervention [31]. Given recent successes and failures [32,33] of adherence-enhancing interventions, further research aimed at improving adherence (through refined adherence intervention strategies and approaches to assessing effectiveness of interventions), will be essential to fully realise the benefits of ART. In the meantime, simple procedures can measure adherence and poor adherence has again clearly been shown to be associated with mortality: lives gained from effective interventions to improve adherence would be considerable. Actively seeking high risk patients who miss clinic appointments might have a significant impact on mortality or at least should be studied rigorously.

## Competing interests

The authors declare that they have no competing interests.

## Authors’ contributions

SK, HO, ASW, PI, JL and JT designed the study. MI, RA and MP collected data. SK, PI did the analyses and wrote the first draft together with HO, ASW, JL and JT. All authors contributed to interpretation of the data, reviewed the manuscript critically and approved the final version.

## Pre-publication history

The pre-publication history for this paper can be accessed here:

http://www.biomedcentral.com/1471-2334/13/395/prepub

## Supplementary Material

Additional file 1 **Appendix A: dynamic logistic regression model.** This file contains detailed descriptions of the dynamic logistic regression model parameters, assumptions and how the model is fitted and references for the model. Click here for file

Additional file 2 **Appendix B: population attributable fraction.** This section contains the estimation procedure of the population attributable fraction as well as the description, including references and illustration of how to build the model (Figure 1). Click here for file
