# Supplementary material for: Dynamic logistic regression model and population attributable fraction to investigate the association between adherence, missed visits and mortality: a study of HIV-infected adults surviving the first year of ART
Source: BMC Infect Dis. 2013 Aug 27;13:395. doi: 10.1186/1471-2334-13-395 (PMC3847061; doi:10.1186/1471-2334-13-395)
Supplement: Additional file 1 — Appendix A: dynamic logistic regression model. This file contains detailed descriptions of the dynamic logistic regression model parameters, assumptions and how the model is fitted and references for the model. [file 1471-2334-13-395-S1.pdf]

Specifically, let, T_i be the total planned number of clinic visits during the follow-up for patient i, $i = 1, ..., n$. The response y_{it} is a binary outcome which indicates whether patient i has died before the tth visit. Let t_i be the observed follow-up time for the ith patient: If patient i does not die during the full follow-up period then $t_i = T_i$, otherwise $t_i = \min \{t : y_{it} = 1\}$. Let x_{it} be the time-dependent vector of explanatory variables for y_{it}, $t = 1, ..., t_i$. (The first component of x_{it} can be assumed to be one for the model intercept.) In a dynamic logistic model we assume that the probability of $y_{it} = 1$ conditional on $y_{i,t-1} = 0$ is

$$r_{it}(\beta) = \left(1 + \exp(-\beta^T x_{ij})\right)^{-1}.$$

Then the likelihood function based on the full data is

$$L(\beta) = \prod_{i=1}^{n} \prod_{t=1}^{t_i} r_{it}^{y_{it}} (1 - r_{it})^{1-y_{it}}.$$

The model can be fitted using standard logistic regression algorithms to give the maximum likelihood (ML) estimate $\hat{\beta}$ with its estimated covariance matrix; we used R software and the glm function with the logit link. The GEE for a vector of Bernoulli responses $(y_{i1}, ..., y_{it_i})$ (for death) cannot be applied here as (i) the length of the vector is varying (random), and the observed vectors are of type 1, (0,1), (0,0,1), ... so to build a correlation structure for these types of observations may be insufficient. Instead, we build the model using conditional probabilities $P(y_{it} = 1 | y_{i1} = ... y_{i,t-1} = 0)$. Standard arguments can be used to show the limiting multivariate normality of $\hat{\beta}$.

The dynamic logistic regression model was first proposed by Bonney et al. [1] and applied by Alho et al. [2] in assessing the monthly risks of middle ear infection. Here we use dynamic logistic regression model to investigate the relationship between adherence to ART and mortality. The estimated odds ratios are for the risk of dying in the current 4-weekly interval. As the time-dependent variables are measured at the clinic visits, these provide a natural discretization for time to death without much loss of information. With this model it is also easy to construct an estimate for the population attributable fraction (PAF).

References

[1] Bonney GE: **Logistic regression for dependent binary observations**. *Biometrics* 1987, **43**(4):951–973.

[2] Alho OP, Läärä E, Oja H: **Public health impact of various risk factors for acute Otitis Media in northern Finland**. *Am. J. Epidemiol.* 1996, **143**(11):1149–1156.
