# Supplementary material for: Dynamic logistic regression model and population attributable fraction to investigate the association between adherence, missed visits and mortality: a study of HIV-infected adults surviving the first year of ART
Source: BMC Infect Dis. 2013 Aug 27;13:395. doi: 10.1186/1471-2334-13-395 (PMC3847061; doi:10.1186/1471-2334-13-395)
Supplement: Additional file 2 — Appendix B: population attributable fraction. This section contains the estimation procedure of the population attributable fraction as well as the description, including references and illustration of how to build the model (Figure 1). [file 1471-2334-13-395-S2.pdf]

If the full covariate history $x_i = (x_{i1}, ..., x_{iT_i})^T$ of individual i is known, then the individual (expected) survival function is

$$S_{it}(\beta) = \prod_{j=1}^{t} (1 - r_{ij}(\beta)).$$

Then the theoretical population survival function for a population with covariate histories given in $X = \{x_1, ..., x_n\}$ is

$$S_t(\beta, X) = \frac{1}{n} \sum_{i=1}^{n} S_{it}(\beta)$$

and $S_t(\beta, X)$ gives the expected proportion of individuals alive at visit t, $t = 1, 2, ...$ If the covariate histories in $X = \{x_1, ..., x_n\}$ are changed to take the values in $X^* = \{x_1^*, ..., x_n^*\}$, then the population survival curve $S_t(\beta, X)$ will transform to $S_t(\beta, X^*)$, and the ratio

$$PAF_t = \frac{S_t(\beta, X^*) - S_t(\beta, X)}{1 - S_t(\beta, X)},$$

the population attributable fraction, gives the (theoretical) proportion of deaths which could have been avoided with the manipulation by time $t = 1, 2, ...$ Naturally, PAF_t can be estimated by

$$\widehat{PAF}_t = \frac{S_t(\hat{\beta}, X^*) - S_t(\hat{\beta}, X)}{1 - S_t(\hat{\beta}, X)},$$

and the delta method can be used to construct limiting confidence intervals, $t = 1, 2, ...$ [1, 2].

The maximum likelihood estimation of attributable fractions was first proposed by Greenland and Drescher [3] in conventional logistic regression framework. An extension to the dynamic logistic regression models was proposed by Oja et al. [2] and public health impact was demonstrated using data on the risk of middle ear infections [4].

Here, we are interested in the impact of adherence behaviour on the risk of death. The covariate vector x_i can then be divided into two subvectors x_{i1} and x_{i2} such that $x_i = (x_{i1}^T, x_{i2}^T)^T$, and x_{i1} includes the adherence variables and x_{i2} the other covariates (confounders). (Figure 1). For the population attributable fraction, the manipulation, is then

$$x_i = \begin{pmatrix} x_{i1} \\ x_{i2} \end{pmatrix} \quad \rightarrow \quad x_i^* = \begin{pmatrix} x_{i1}^* \\ x_{i2} \end{pmatrix}$$

In order to estimate the population attributable fraction due to non-optimal adherence, x_{i1} will be set to x_{i1}^* (meaning optimal adherence) while x_{i2} will be left unchanged. Note that, in our case, the full adherence history of patient i who dies is not known (up to visit T_i) so that $S_t(\hat{\beta}, X)$ cannot be calculated directly. We therefore compare the estimated (Kaplan-Maier) population survival curve from the original data (excluding STI patients after STI randomisation and upweighting equivalent patients randomised to CT), and the curve $S_t(\hat{\beta}, X^*)$ to find an estimate for PAF_t, $t = 1, 2, ...$ For confidence intervals, $M = 200$ bootstrap samples were generated from our 2960 patients with resulting estimates $\widehat{PAF}_{t,m}$, $m = 1, ..., M$, $t = 1, 2, ...$. The bootstrap estimates $\widehat{PAF}_{t.m}$ are then used to calculate 90% confidence intervals for PAF_t, $t = 1, 2, ...$. Note that, if the lower limit of the confidence interval is positive, then a one-sided level 0.05 test rejects the null hypothesis "the manipulation has no effect". We also prefer 90% confidence interval here as M should be much larger for an accurate estimation of 95% confidence interval.

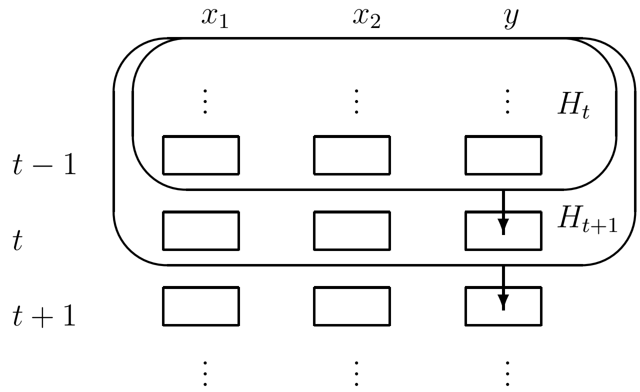

Figure 1: Illustration of the dynamic model: The probability $P(y_t = 1 | y_1 = ..., y_{t-1} = 0)$ is modeled using history H_t, that is, the values $x_{1,1}, ..., x_{1,t-1}$ and $x_{2,1}, ..., x_{2,t-1}$.

References

[1] Leung HM, Kupper LL: **Comparisons of confidence intervals for attributable risk**. *Biometrics* 1981, **37**(2):293–302.

[2] Oja H, Alho OP, Läärä E: **Model-based estimation of the excess fraction (attributable fraction): day care and middle ear infection**. *Statistics in Medicine* 1996, **15**(2):1519–1534.

[3] Greenland S, Drescher K: **Maximum likelihood estimation of the attributable fraction from logistic models**. *Biometrics* 1993, **49**(3):865–872.

[4] Alho OP, Läärä E, Oja H: **Public health impact of various risk factors for acute Otitis Media in northern Finland**. *Am. J. Epidemiol.* 1996, **143**(11):1149–1156.
